# Circular Dichroism Second-Harmonic Generation Imaging of KTiOPO_4_ Nanocrystal Through Stratified Media

**DOI:** 10.3389/fchem.2022.845311

**Published:** 2022-04-08

**Authors:** Biwei Wu, Keyi Wu, Xuefeng Sun, Weibo Wang, Jiubin Tan

**Affiliations:** ^1^ Institute of Ultra-precision Optoelectronic Instrument Engineering, Harbin Institute of Technology, Harbin, China; ^2^ Key Lab of Ultra-precision Intelligent Instrumentation, Harbin Institute of Technology, Ministry of Industry and Information Technology, Harbin, China

**Keywords:** nanocrystal, nonlinear optics, second-harmonic generation, polarization, imaging

## Abstract

Potassium titanyl phosphate (KTiOPO_4,_ KTP) particle of nanometric size (nano-KTP) is an attractive material for nonlinear microscopy, and the optimized growth of large-size KTP single crystals has numerous applications for efficient frequency conversion in laser technology. Its three-dimensional orientation and nanoscale morphology are important for growth optimization. In this paper, we introduce an imaging technique based on circular dichroism second-harmonic generation (CD-SHG) to characterize the 3D distribution of KTP nanocrystal. A rigorous theoretical model of CD-SHG imaging for nano-KTP through stratified media is demonstrated. Circular dichroism analysis is used to probe the orientation of 3-axis with respect to the optical observation axis. The research results show that the azimuthal angle of the peak value (SHG) or valley value (CD-SHG) is strongly related to the excitation polarization when the KTP sample is excited by different circular polarizations. Importantly, the refractive index mismatches and the imaging depth also affect the azimuthal angle. Thus, the proposed framework enables a more precise quantitative analysis of the CD-SHG signal of KTP.

## Introduction

Potassium titanyl phosphate (KTiOPO_4_; KTP) has been widely used in several nonlinear-optical applications, including parametric generation and amplification, by virtue of its superior performance in nonlinear-optical coefficients, large acceptance angles, high optical damage threshold, and thermally stable phase-matching properties (Bierlein and Vanherzeele, 1989). In particular, the optimized growth of large-size single KTP crystals plays an important role for efficient frequency conversion in laser technology (Driscoll et al., 1986). Recently, second harmonic generation (SHG) in diamond-blade diced KTP ridge waveguides has also been demonstrated (Chen et al., 2016). Furthermore, KTP particle of nanometric size (nano-KTP) is a suitable nonlinear crystal material for SHG microscopy, which can generate a stable blinking-free second-harmonic signal that can be easily detected (Le Xuan et al., 2008). Notably, the three-dimensional orientation and nanoscale morphology of KTP are important for growth optimization.

In a SHG process, two photons of frequency ω absorbed by the ground state combine to form a doubled frequency (2ω) photon. SHG-based techniques have been applied in the investigation of semi-conductor nanowires with different composition and nanoscale morphology detection of nano-objects (Bautista et al., 2015; Ren et al., 2015; Bautista et al., 2012; Bautista et al., 2017). Importantly, polarization-resolved SHG microscopy has proven to be an effective all-optical mode for *in situ* measurement of underlying crystal structures without sample damages, as SHG is sensitive to the polarization of excitation fields (Gleeson et al., 2020). Moreover, SHG circular dichroism (CD) parameter has been recently developed to explicitly evidence the presence of a chiral response of the nanocrescents induced by the geometry of hybrid plasmonic–photonic nanosurfaces (Belardini et al., 2014). However, the potential of CD-SHG microscopy to probe three-dimensional orientation of KTP with respect to the optical observation axis and the polarity distribution of out-of-plane nanocrystal assemblies has not been explored.

It is worth noting that in polarization-resolved SHG microscopy for crystal material, the specimen is often observed with a high NA objective with immersion medium and a cover glass. Moreover, they usually have different refractive indices. For example, the refractive index of KTP is 1.738 (1,064 nm) (Sutherland, 2003), which is much larger than that of immersion medium or cover glass. When the excitation beams are focused through the stratified media, aberrations are introduced due to the refractive index mismatches. The presence of the aberration will cause a structural and polarization state modification of the focused spot, and then lead to a performance degradation of the polarization-resolved SHG microscopy. However, the currently used theoretical frameworks are inapplicable for the analysis of polarization-resolved SHG imaging, which are based on the assumption that the specimen is situated in a homogeneous medium of propagation or located at a dielectric interface, neglecting the influence of sample refractive index heterogeneity near the focus. As a complementary tool with a different principle, the finite-difference time-domain (FDTD) approach has been adopted in many microscopy technique simulations, such as wide-field, confocal, and SHG microscopy (Török et al., 2008; Choi et al., 2007; van der Kolk et al., 2018). More recently, the ubiquitous geometry of a vertical interface between index-mismatched media and the case of polarized THG contrasts are analyzed based on FDTD methods (Morizet et al., 2021). The effects of the refractive index mismatches and the imaging depth on CD-SHG microscopy have not yet been studied.

Here, we present a rigorous theoretical framework of CD-SHG microscopy through stratified media for KTP crystal. The SHG intensity patterns for left-handed and right-handed circular polarized excitations and the corresponding CD-SHG signal obtained in a mismatch free medium and in a mismatched stratified media will be compared from several aspects. The quantitative analysis of azimuthal angle of SHG signal and the SHG response to a specific point object as a function of the effective NA in different depths will be demonstrated. In addition, circular-polarization-excited SHG imaging in index-mismatched media in the case of a vertical interface between water and a KTP material are investigated based on FDTD methods. As a complementary polarization-resolved scheme, CD-SHG imaging not only provides intrinsic 3D imaging capabilities with sub-micrometer spatial resolution, but also paves the way for determining the local 3D orientation of KTP molecules information with a new structural contrast mechanism. We also expect that the study can contribute towards new insights into optimization of CD-SHG microscopy instrumentation.

## Modeling of Circular Dichroism Second-Harmonic Generation Through Stratified Media

**FIGURE 1:**
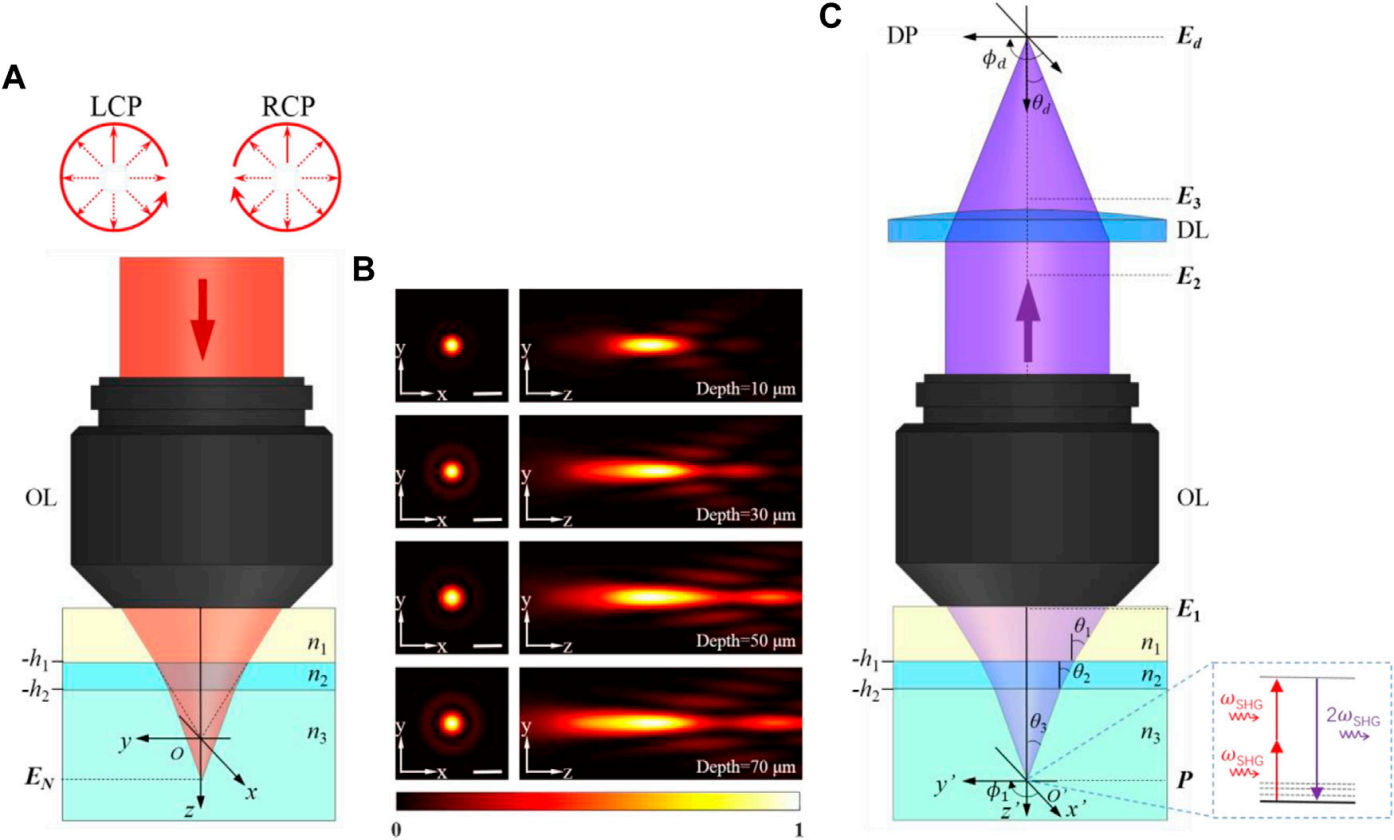
**(A)** Schematic of focusing through a three-layer stratified medium for left-handed and right-handed circular polarized beams. The origin *O* of the (*x*, *y*, *z*) reference frame locates at the nominal focal point position. **(B)** Near-focus excitation intensity distributions at different depths. **(C)** SHG radiation imaged through the same medium. The specimen is placed at the origin *O’* of the (*x’*, *y’*, *z’*) reference frame. LCP: left-handed circular polarization, RCP: right-handed circular polarization, OL: objective lens, DP: detector plane, DL: detector lens. Scale bar, 1 μm.

Figure 1 shows the most common geometry of a polarization-resolved SHG microscope, including the configuration for excitation by left-handed and right-handed circular polarized beams through a high NA objective and a three-layer medium. The specimen is observed through an immersion medium and a coverslip. The first interface, perpendicular to the optical axis, is at *z* = −*h*_1_, and the second at *z* = −*h*_2_. The wave numbers of the light beam in the immersion medium, cover glass and specimen are *k*_1_, *k*_2_, and *k*_3_, respectively. *k*_1_ = 2π*n*_1_/*λ*_0_, *k*_2_ = 2π*n*_2_/*λ*_0_, and *k*_3_ = 2π*n*_3_/*λ*_0_. *n*_1_, *n*_2_, and *n*_3_ are the refractive index of the immersion medium, cover glass, and specimen respectively.

Based on the Richards–Wolf vectorial diffraction integral (Richards and Wolf, 1959; Török and Varga, 1997; Haeberlé et al., 2003), the generalized formulae for the vectorial electric field in the focal region illuminated by left-handed and right-handed circular polarized beams through an *N*-layer medium are derived. Under the idiomatic polar coordinate system notation, the Cartesian components of the electric field in the focal region can be expressed as:

$$E_{Nx}=A\left[I_{cir-0}^{\left(N\right)}+I_{cir-2}^{\left(N\right)}\exp\left(\pm 2i\phi_{P}\right)\right],$$ (1)

$$E_{Ny}=iA\left[I_{cir-0}^{\left(N\right)}-I_{cir-2}^{\left(N\right)}\exp\left(\pm 2i\phi_{P}\right)\right],$$ (2)

$$E_{Nz}=-2iAI_{cir-1}^{\left(N\right)}\exp\left(\pm i\phi_{P}\right),$$ (3)

and the integrals $I_{cir-0}^{\left(N\right)}$ , $I_{cir-1}^{\left(N\right)}$ , and $I_{cir-2}^{\left(N\right)}$ are given by

$$\begin{array}{l} I_{cir-0}^{\left(N\right)}=\int_{0}^{\alpha_{1}}\cos^{1/2}\theta_{1}\sin\theta_{1}\exp\left(ik_{0}\psi_{i}\right)\left(T_{s}^{\left(N-1\right)}+T_{p}^{\left(N-1\right)}\cos\theta_{N}\right)\\ \times J_{0}\left(k_{1}r_{P}\sin\theta_{1}\sin\theta_{P}\right)\exp\left(ik_{N}r_{P}\cos\theta_{N}\cos\theta_{P}\right)d\theta_{1}, \end{array}$$ (4)

$$\begin{array}{l} I_{cir-1}^{\left(N\right)}=\int_{0}^{\alpha_{1}}\cos^{1/2}\theta_{1}\sin\theta_{1}\exp\left(ik_{0}\psi_{i}\right)T_{p}^{\left(N-1\right)}\sin\theta_{N}\\ \times J_{1}\left(k_{1}r_{P}\sin\theta_{1}\sin\theta_{P}\right)\exp\left(ik_{N}r_{P}\cos\theta_{N}\cos\theta_{P}\right)d\theta_{1}, \end{array}$$ (5)

$$\begin{array}{l} I_{cir-2}^{\left(N\right)}=\int_{0}^{\alpha_{1}}\cos^{1/2}\theta_{1}\sin\theta_{1}\exp\left(ik_{0}\psi_{i}\right)\left(T_{s}^{\left(N-1\right)}-T_{p}^{\left(N-1\right)}\cos\theta_{N}\right)\\ \times J_{2}\left(k_{1}r_{P}\sin\theta_{1}\sin\theta_{P}\right)\exp\left(ik_{N}r_{P}\cos\theta_{N}\cos\theta_{P}\right)d\theta_{1}, \end{array}$$ (6)

$$\psi_{i}=h_{N-1}n_{N}s_{Nz}-h_{1}n_{1}\cos\theta_{1}s_{1z},$$ (7)

where (*r*_*P*_, *θ*_*P*_, *ϕ*_*P*_) are the spherical polar coordinates of an observation point *P* near the focal region, respectively. *α*_1_ is the convergence semi-angle of the illumination, and is given as *α*_1_ = arcsin (NA/*n*1). *l*_0_ (*θ*_1_) is the amplitude function in terms of *θ*_1_ and *J*_n_(*x*) denotes a Bessel function of the first kind, of order *n*. $T_{p}^{\left(N-1\right)}$ and $T_{s}^{\left(N-1\right)}$ are the transmission coefficient of the stratified medium describing the *p*- and *s*-polarized light traversing *N*−1 media respectively and calculated as in Török and Varga (1997). *ψ*_*i*_ denotes the initial aberration function. Figure 1B shows the near-focus excitation intensity distributions at different depths.

As the focal electric field distribution is determined, the interaction with the material can be calculated. SHG intensity has a quadratic relationship with the optical field intensity of the excitation beam at the focus region, which is also dependent on the nonlinear susceptibility tensor of the sample. When the frequency of the laser light source is far away from the resonant frequency of the specimen, the number of non-zero elements in this third-order tensor with 27 separate elements reduces according to Kleinmann’s symmetry (Kleinman, 1962; Yew and Sheppard, 2006). SHG polarization is related to the focused laser excitation field by:

$$\left[\begin{array}{c} P_{x}\\ P_{y}\\ P_{z} \end{array}\right]=\left[\begin{array}{cccccc} d_{xxx} & d_{xyy} & d_{xzz} & d_{xyz} & d_{xxz} & d_{xxy}\\ d_{yxx} & d_{yyy} & d_{yzz} & d_{yyz} & d_{yxz} & d_{yxy}\\ d_{zxx} & d_{zyy} & d_{zzz} & d_{zyz} & d_{zxz} & d_{zxy} \end{array}\right]\left[\begin{array}{c} E_{Nx}E_{Nx}\\ E_{Ny}E_{Ny}\\ E_{Nz}E_{Nz}\\ 2E_{Ny}E_{Nz}\\ 2E_{Nx}E_{Nz}\\ 2E_{Nx}E_{Ny} \end{array}\right].$$ (8)

The SHG polarization emits corresponding radiation, and the harmonic field ***E***_**1**_ traverses back the stratified medium (Figure 1C). The distribution before the objective lens (in medium 1) can be expressed as (Török, 2000):

$$\begin{array}{l} E_{1x^{\prime }}=\frac{1}{2}[P_{x}^{*}\left(T_{s}^{\prime }+T_{p}^{\prime }\cos\theta_{N}\cos\theta_{1}\right)-2P_{z}^{*}T_{p}^{\prime }\sin\theta_{N}\cos\theta_{1}\cos\phi_{1}\\ -{\left(T_{s}^{\prime }-T_{p}^{\prime }\cos\theta_{N}\cos\theta \right)}_{1}\left(P_{x}^{*}\cos2\phi_{1}+P_{y}^{*}\sin2\phi_{1}\right)], \end{array}$$ (9)

$$\begin{array}{l} E_{1y^{\prime }}=\frac{1}{2}[P_{y}^{*}\left(T_{s}^{\prime }+T_{p}^{\prime }\cos\theta_{N}\cos\theta_{1}\right)-2P_{z}^{*}T_{p}^{\prime }\sin\theta_{N}\cos\theta_{1}\sin\phi_{1}\\ -\left(T_{s}^{\prime }-T_{p}^{\prime }\cos\theta_{N}\cos\theta_{1}\right)\left(P_{x}^{*}\sin2\phi_{1}-P_{y}^{*}\cos2\phi_{1}\right)], \end{array}$$ (10)

$$E_{1z^{\prime }}=\left[P_{z}^{*}T_{p}^{\prime }\sin\theta_{N}\sin\theta_{1}-T_{p}^{\prime }\cos\theta_{N}\sin\theta_{1}\left(P_{x}^{*}\cos\phi_{1}+P_{y}^{*}\sin\phi_{1}\right)\right].$$ (11)

(*P*_x_^∗^, *P*_y_^∗^, *P*_z_^∗^) denotes the Cartesian components of the complex conjugate of ***P***. The transmission coefficients *T*_*s*_ *’* and *T*_*p*_ *’* for the stratified medium can be calculated as in Haeberlé et al. (2003) and Török and Varga (1997), but with propagation direction from medium *N* to medium 1.

After being collimated by the objective lens, the Cartesian components of the harmonic field vector ***E***_**2**_ in the intermediate plane is given by (Wang et al., 2019):

$$\begin{array}{l} E_{2x^{\prime }}={\left(\cos\theta_{1}\right)}^{-1/2}\{P_{x}^{*}\left(T_{p}^{\prime }\cos^{2}\phi_{1}\cos\theta_{N}+T_{s}^{\prime }\sin^{2}\phi_{1}\right)\\ +P_{y}^{*}\left(T_{p}^{\prime }\cos\theta_{N}-T_{s}^{\prime }\right)\sin\phi_{1}\cos\phi_{1}-P_{z}^{*}T_{p}^{\prime }\sin\theta_{N}\cos\phi_{1}\}, \end{array}$$ (12)

$$\begin{array}{l} E_{2y^{\prime }}={\left(\cos\theta_{1}\right)}^{-1/2}\{P_{x}^{*}\cos\phi_{1}\left(T_{p}^{\prime }\cos\theta_{N}-T_{s}^{\prime }\right)\sin\phi_{1}\\ +P_{y}^{*}\left(T_{s}^{\prime }\cos^{2}\phi_{1}+T_{p}^{\prime }\sin^{2}\phi_{1}\cos\theta_{N}\right)-P_{z}^{*}T_{p}^{\prime }\sin\phi_{1}\sin\theta_{N}\}, \end{array}$$ (13)

Next, the field vector ***E***_**3**_, behind the detector lens can be described by

$$\begin{array}{l} E_{3x}={\left(\cos\theta_{d}\right)}^{1/2}{\left(\cos\theta_{1}\right)}^{-1/2}\{P_{x}^{*}\left[\frac{1}{2}T_{s}^{\prime }\left(1-\cos2\phi_{1}\right)+\frac{1}{2}T_{p}^{\prime }\cos\theta_{d}\cos\theta_{N}\left(1+\cos2\phi_{1}\right)\right]\\ +P_{y}^{*}\left[\frac{1}{2}T_{p}^{\prime }\cos\theta_{d}\cos\theta_{N}-\frac{1}{2}T_{s}^{\prime }\right]\sin2\phi_{1}-P_{z}^{*}T_{p}^{\prime }\cos\theta_{d}\sin\theta_{N}\cos\phi_{1}\}, \end{array}$$ (14)

$$\begin{array}{l} E_{3y}={\left(\cos\theta_{d}\right)}^{1/2}{\left(\cos\theta_{1}\right)}^{-1/2}\{P_{x}^{*}\left[\frac{1}{2}\left(-T_{s}^{\prime }+T_{p}^{\prime }\cos\theta_{d}\cos\theta_{N}\right)\sin2\phi_{1}\right]\\ +P_{y}^{*}\left[\frac{1}{2}T_{s}^{\prime }\left(1+\cos2\phi_{1}\right)+\frac{1}{2}T_{p}^{\prime }\cos\theta_{d}\cos\theta_{N}\left(1-\cos2\phi \right)\right]-P_{z}^{*}T_{p}^{\prime }\cos\theta_{d}\sin\theta_{N}\sin\phi_{1}\}, \end{array}$$ (15)

$$\begin{array}{l} E_{3z}={\left(\cos\theta_{d}\right)}^{1/2}{\left(\cos\theta_{1}\right)}^{-1/2}\{P_{x}^{*}\left(-T_{p}^{\prime }\sin\theta_{d}\cos\theta_{N}\cos\phi_{1}\right)\\ +P_{y}^{*}\left(-T_{p}^{\prime }\sin\theta_{d}\cos\theta_{N}\sin\phi_{1}\right)+P_{z}^{*}T_{p}^{\prime }\sin\theta_{d}\sin\theta_{N}\}. \end{array}$$ (16)

Hence, the expression for the SHG field components at the detector plane can be obtained by using the integral formula of Richards and Wolf (Richards and Wolf, 1959; Török et al., 1998), as:

$$E_{dx}=-iA_{d}P_{x}^{*}I_{d01}+iA_{d}P_{x}^{*}I_{d21}\cos2\phi_{d}+iA_{d}P_{y}^{*}I_{d21}\sin2\phi_{d}-2A_{d}P_{z}^{*}I_{d11}\cos\phi_{d},$$ (17)

$$E_{dy}=iA_{d}P_{x}^{*}I_{d21}\sin2\phi_{d}-iA_{d}P_{y}^{*}I_{d01}-iA_{d}P_{y}^{*}I_{d21}\cos2\phi_{d}-2A_{d}P_{z}^{*}I_{d11}\sin\phi_{d},$$ (18)

$$E_{dz}=-2A_{d}P_{x}^{*}I_{d12}\cos\phi_{d}-2A_{d}P_{y}^{*}I_{d12}\sin\phi_{d}-2iA_{d}P_{z}^{*}I_{d02},$$ (19)

with the quantities *I*_*d*01_, *I*_*d*02_, *I*_*d*11_, *I*_*d*12_, and *I*_*d*21_ defined as:

$$\begin{array}{l} I_{d01}=\int_{0}^{\alpha_{d}}{\left(\cos\theta_{d}\right)}^{1/2}{\left(\cos\theta_{1}\right)}^{-1/2}\left(T_{s}^{\prime }\sin\theta_{d}+T_{p}^{\prime }\sin\theta_{d}\cos\theta_{d}\cos\theta_{N}\right)J_{0}\left(k_{d}\rho_{d}\sin\theta_{d}\right)\\ \times \exp\left(-ik_{d0}\psi_{\det}\right)\exp\left(-ik_{d}z_{d}\cos\theta_{d}\right)d\theta_{d}, \end{array}$$ (20)

$$\begin{array}{l} I_{d02}=\int_{0}^{\alpha_{d}}{\left(\cos\theta_{d}\right)}^{1/2}{\left(\cos\theta_{1}\right)}^{-1/2}\left(T_{p}^{\prime }\sin^{2}\theta_{d}\sin\theta_{N}\right)J_{0}\left(k_{d}\rho_{d}\sin\theta_{d}\right)\\ \times \exp\left(-ik_{d0}\psi_{\det}\right)\exp\left(-ik_{d}z_{d}\cos\theta_{d}\right)d\theta_{d}, \end{array}$$ (21)

$$\begin{array}{l} I_{d11}=\int_{0}^{\alpha_{d}}{\left(\cos\theta_{d}\right)}^{1/2}{\left(\cos\theta_{1}\right)}^{-1/2}\left(T_{p}^{\prime }\cos\theta_{d}\sin\theta_{d}\sin\theta_{N}\right)J_{1}\left(k_{d}\rho_{d}\sin\theta_{d}\right)\\ \times \exp\left(-ik_{d0}\psi_{\det}\right)\exp\left(-ik_{d}z_{d}\cos\theta_{d}\right)d\theta_{d}, \end{array}$$ (22)

$$\begin{array}{l} I_{d12}=\int_{0}^{\alpha_{d}}{\left(\cos\theta_{d}\right)}^{1/2}{\left(\cos\theta_{1}\right)}^{-1/2}\left(T_{p}^{\prime }\sin^{2}\theta_{d}\cos\theta_{N}\right)J_{1}\left(k_{d}\rho_{d}\sin\theta_{d}\right)\\ \times \exp\left(-ik_{d0}\psi_{\det}\right)\exp\left(-ik_{d}z_{d}\cos\theta_{d}\right)d\theta_{d}, \end{array}$$ (23)

$$\begin{array}{l} I_{d21}=\int_{0}^{\alpha_{d}}{\left(\cos\theta_{d}\right)}^{1/2}{\left(\cos\theta_{1}\right)}^{-1/2}\left(-T_{s}^{\prime }\sin\theta_{d}+T_{p}^{\prime }\sin\theta_{d}\cos\theta_{d}\cos\theta_{N}\right)\\ \times J_{2}\left(k_{d}\rho_{d}\sin\theta_{d}\right)\cdot \exp\left(-ik_{d0}\psi_{\det}\right)\exp\left(-ik_{d}z_{d}\cos\theta_{d}\right)d\theta_{d}, \end{array}$$ (24)

*α*_*d*_ is the angular aperture of the detector lens. *ρ*_*d*_, *ϕ*_*d*_, and *z*_*d*_ are the cylindrical coordinates of an observation point near the detection region and the azimuthal angle *θ*_*d*_ is related to the azimuthal angle *θ*_1_ by the relationship:

$$\frac{k_{d1}\sin\alpha_{1}}{k_{d}\sin\alpha_{d}}=\frac{k_{d1}\sin\theta_{1}}{k_{d}\sin\theta_{d}}=M,$$ (25)

where *M* is the magnification of the imaging system. *k*_*d*0_, *k*_*d*1_, and *k*_*d*_ are the wave numbers for the second-harmonic field in vacuum, medium 1 (immersion medium), and the image space, respectively, expressed as *k*_*d*0_ = 2π/*λ*_SHG_, *k*_*d*1_ = 2π*n*_1_/*λ*_SHG_, and *k*_*d*_ = 2π*n*_*d*_/*λ*_SHG_. *λ*_SHG_ denotes the wavelength of SHG. *n*_*d*_ is the refractive index of the image space. The aberration function in the detection path *ψ*_det_ can be expressed by

$$\Psi_{\det}=n_{1}h_{1}\cos\theta_{1}-n_{N}h_{N-1}\cos\theta_{N}.$$ (26)

As a result, the detected SHG intensity excited by left-handed and right-handed circular polarized beams through stratified media can be obtained according to:

$$I_{SHG}={\left|E_{dx}\right|}^{2}+{\left|E_{dy}\right|}^{2}+{\left|E_{dz}\right|}^{2}.$$ (27)

In the end, CD-SHG is measured as the normalized difference between SHG signals $I_{SHG}^{L}$ and $I_{SHG}^{R}$ excited with a left-handed circular and a right-handed circular polarization (Schmeltz et al., 2020):

$$\text{CD}-\text{SHG}=\frac{I_{SHG}^{L}-I_{SHG}^{R}}{\left(I_{SHG}^{L}+I_{SHG}^{R}\right)/2}.$$ (28)

## Results and Discussion

KTP is a non-centrosymmetric orthorhombic crystal with large nonlinear-optical coefficients. The accurate magnitude of the nonlinear optical coefficients *d* (in pm/V) of KTP has been measured (Vanherzeele and Bierlein, 1992), and with the values *d*_*xxz*_ = 1.91, *d*_*yyz*_ = 3.64, *d*_*zxx*_ = 2.54, *d*_*zyy*_ = 4.35, *d*_*zzz*_ = 16.9 when excited in the wavelength of 1,064 nm. It is noteworthy that *d*_*zzz*_ is the dominant nonlinear optical coefficient. The induced SHG polarization can be expressed as:

$$\begin{array}{l} P_{x}=2d_{xxz}E_{Nx}E_{Nz},\\ P_{y}=2d_{yyz}E_{Ny}E_{Nz},\\ P_{z}=d_{zxx}E_{Nx}E_{Nx}+d_{zyy}E_{Ny}E_{Ny}+d_{zzz}E_{Nz}E_{Nz}, \end{array}$$ (29)

**FIGURE 2:**
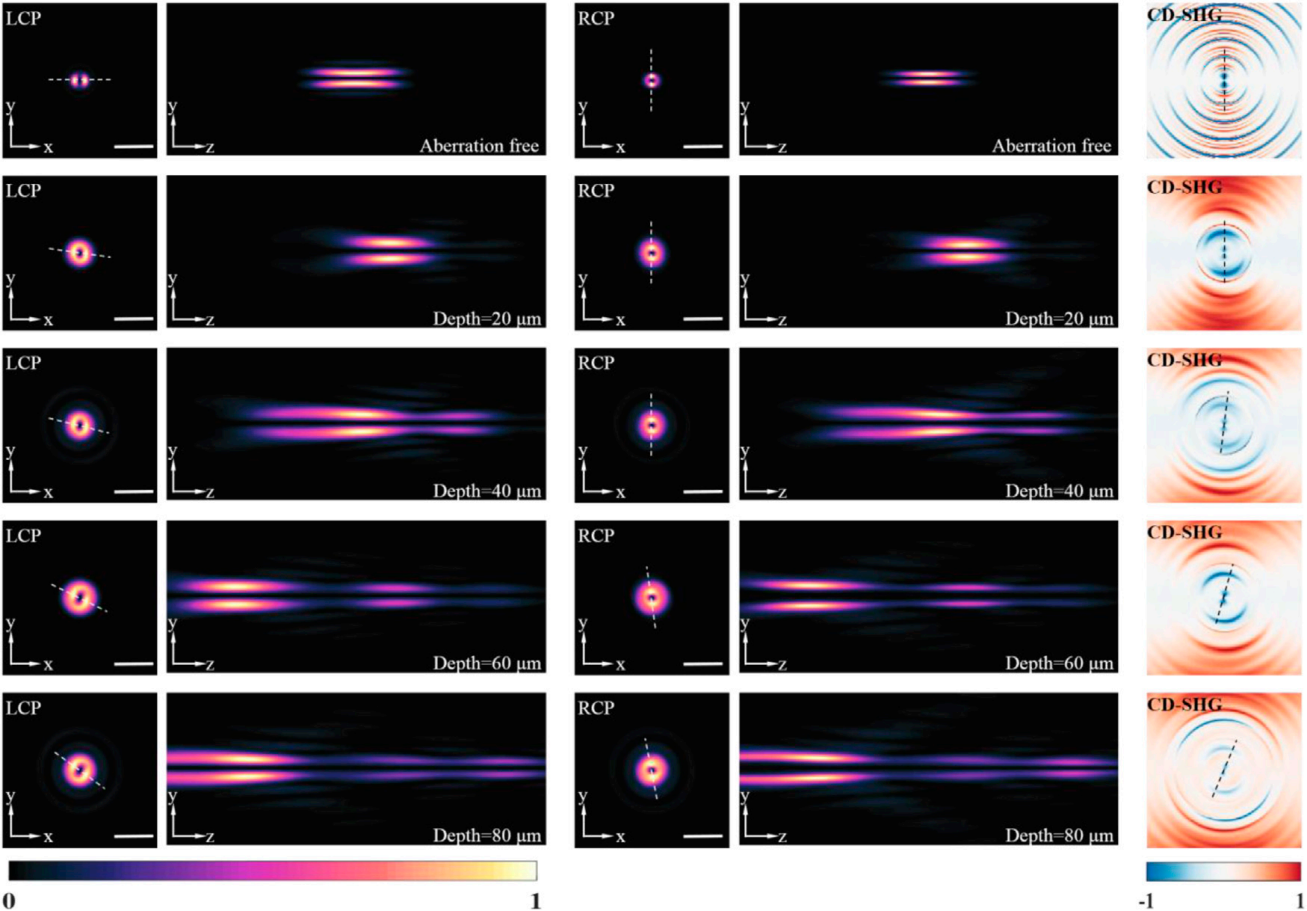
The detected SHG intensity distributions excited by left-handed, right-handed circular polarized beams at different imaging depths, and corresponding CD-SHG signal. All the intensity distributions are normalized by the respective maximum value. Scale bar, 1 μm.

The SHG intensity patterns for a refractive index perfectly matched, aberration-free medium (*n*_1_ = *n*_2_ = *n*_3_) and mismatched stratified media, excited by left-handed and right-handed circular polarized beams at different imaging depths, are shown in Figure 2. For a fair comparison, the calculations are performed at *λ* = 1,064 nm and an oil immersion (*n*_1_ = 1.518) objective of NA = 1.2 is considered. The nominal magnification of the imaging system *M* is set to 100. For the mismatched stratified media, the KTP specimen (*n*_3_ = 1.738) is mounted below a 170-µm cover glass (*n*_2_ = 1.525). The imaging depth (*h*_2_) is set to 20, 40, 60, and 80 µm, respectively. It is easy to observe a variation for the SHG intensity pattern when the KTP sample is excited by different circular polarizations. The azimuthal angle of the peak value (SHG) or valley value (CD-SHG) were calculated, as listed in Table 1. The azimuthal angle of the peak value (SHG) or valley value (CD-SHG) is strongly related to the excitation polarization. Besides, for the same excitation, the refractive index mismatches and the imaging depth also affect the azimuthal angle of the peak value (SHG) or valley value (CD-SHG). On the other hand, the SHG intensity distribution of aberration-free medium is symmetric along the *z*-axis and has no side lobes. The location of the peak intensity is at the nominal focus. In contrast, for the mismatched layered media, there is an aberrational focus shift. As the imaging depth increases, the displacement increases and the stretching of the intensity distribution in the *z*-direction becomes more pronounced. In this context, the three-dimensional orientation of KTP can be probed *via* the sign, azimuthal angle, and CD-SHG amplitude distribution. At the same time, the nanoscale morphology can be revealed by the scanned CD-SHG images.

**TABLE 1:** The azimuthal angle (degree) of the peak value (SHG) or valley value (CD-SHG) in Figure 2.

Depth (μm)	LCP	RCP	CD-SHG
Aberration free	0	90.0	90.0
20	172.9	90.0	90.0
40	166.0	90.0	82.9
60	153.4	97.1	73.3
80	143.1	100.6	66.8

**FIGURE 3:**
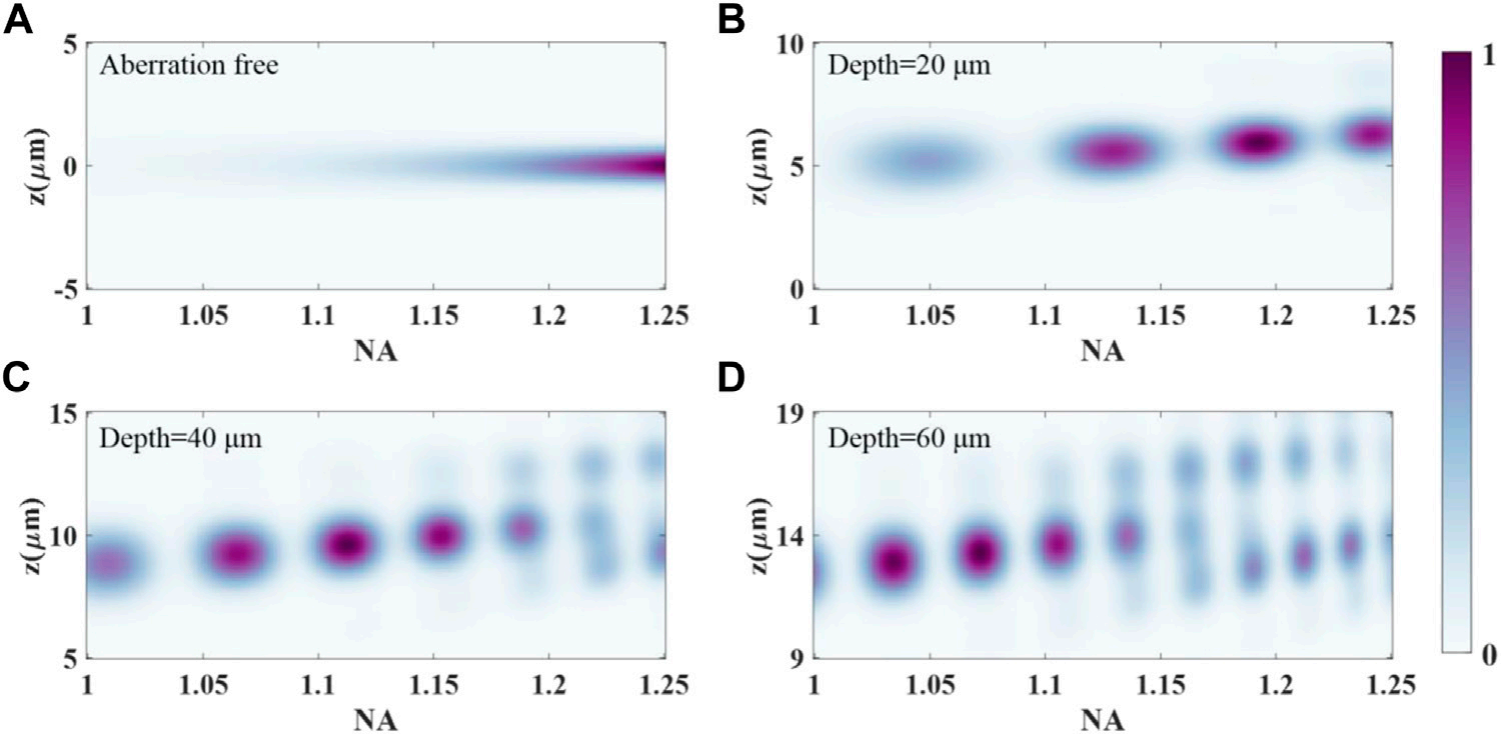
SHG response to a point object located at the coordinate (*x* = 0.14 μm, *y* = 0 μm), excited with a left-handed circular polarization, as a function of the effective NA for an oil immersion objective of maximum NA = 1.25. **(A)** Refractive index mismatch free, **(B)** depth = 20 μm, **(C)** depth = 40 μm, **(D)** depth = 60 µm.

The effects of varying effective NA when focusing to a certain depth in the specimens is essential for the optimization of the experimental polarization-resolved SHG configuration. The variation of effective NA is controlled by changing the pupil size with the iris. The other configuration parameters of the polarization-resolved SHG imaging system are identical to the system described above. Figure 3 shows how the SHG response to a specific point object, excited with a left-handed circular polarization, is affected by altering the pupil size. The point object is located at the coordinate (*x* = 0.14 μm, *y* = 0 μm), which corresponds to the SHG signal peak location of aberration-free medium excited by left-handed circular polarized beams. The distribution along each vertical section shows the axial distribution for a given NA. Each distribution is normalized to the respective maximum intensity. The results for focusing into a perfectly matched, aberration-free medium are shown in Figure 3A. There is a regular shape of the SHG distribution for this case. Figures 3B–D reveal the distributions when focusing to depths of 20, 40, and 60 µm with refractive index mismatch, respectively. As the focusing depth gets to 20 μm, the distribution along the vertical section is broader than that in the aberration-free case, with no significant side lobes. It should also be pointed out that the NA corresponding to the peak intensity changes to 1.19. The shape of the axial distribution degenerates at higher NAs when focusing to a depth of 40 µm. These effects are further exaggerated at an imaging depth of 60 μm, and the axial distribution is severely distorted due to the specimen-induced spherical aberration. There are also significant side lobes along the vertical profile as the effective NA increases. These results are in accordance with the fact that high NA imaging systems are more sensitive to the aberration. In addition, it has been found that right-handed circular polarization has almost the same analysis result with left-handed circular polarization.

**FIGURE 4:**
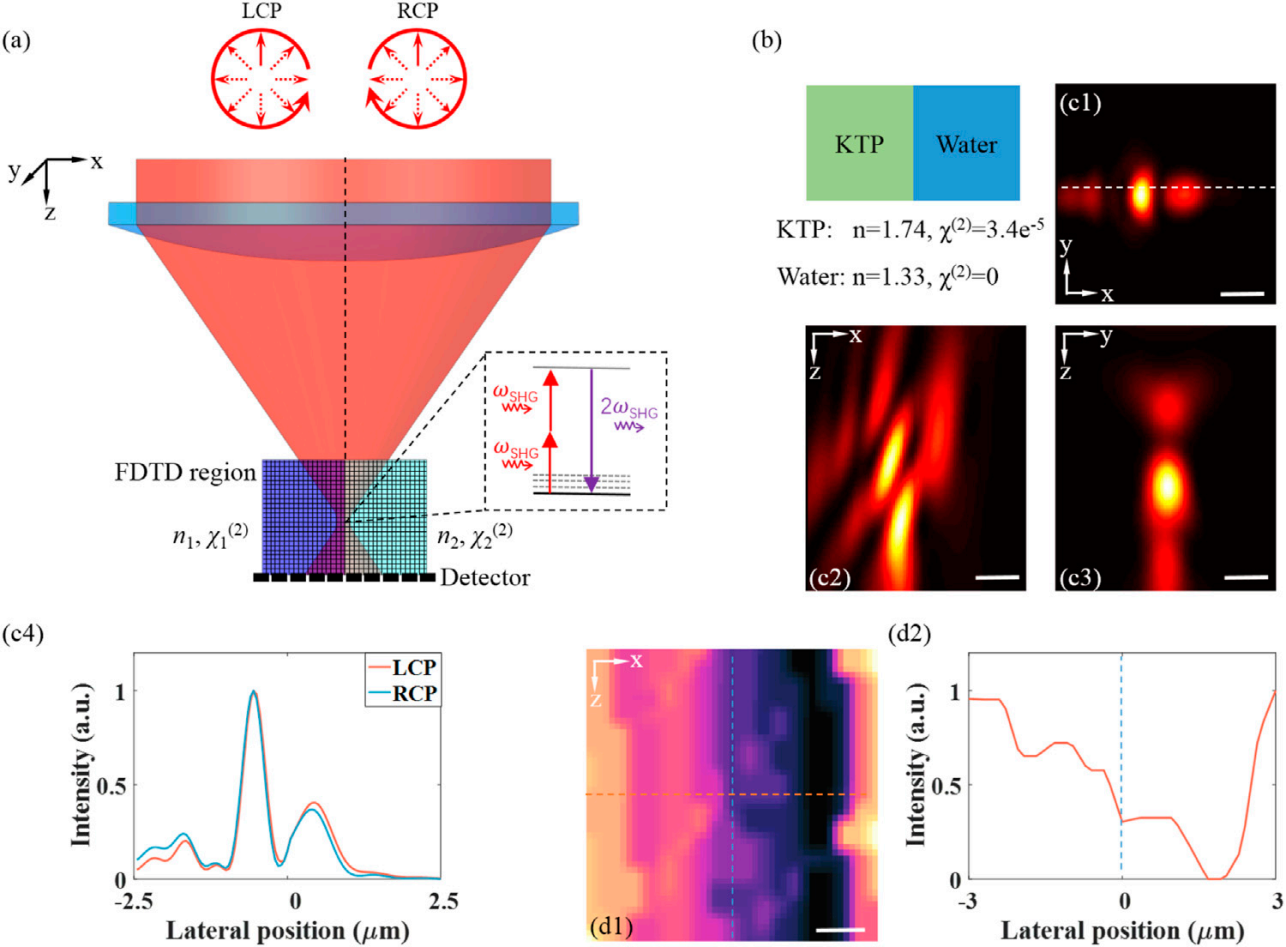
**(A)** Schematic of an FDTD simulation for nonlinear microscopy for under circular polarized excitations to a vertical interface. **(B)** Simplified simulation geometry and properties of the simulation materials. **(C1–C3)** Near-focus excitation intensity distributions along a vertical interface located near the focus for left-handed circular polarized beams. **(C4)** The intensity line profiles along the horizontal lines in **(C1)**. **(D1,D2)** The SHG signal response distribution to a vertical KTP–water interface and corresponding intensity line profile. Scale bar, 1 μm.

For a vertical interface between materials with refractive index mismatches, it has been found that commonly used diffraction-integral-based simulation strategies fail to generate accurate SHG distribution. In contrast, the FDTD family of methods calculate the electric fields at every point of a 3D grid in successive times by solving discretized Maxwell equations for specified materials. The implementation details and the validity of FDTD strategies in the context of nonlinear microscopy have been demonstrated (Morizet et al., 2021). Figure 4A shows the schematic of an FDTD simulation for SHG microscopy under circular polarized excitation to a vertical interface. Here, FDTD calculations are performed firstly to evaluate the field distribution when focused on a vertical KTP–water interface for circular polarized beams. An incoming Gaussian beam with a central wavelength of 1,045 nm and 10-nm bandwidth is tightly focused by an objective lens (NA = 1.0) in the simulation. The calculations are carried out over a focal region spanning 15 μm^3^ × 15 μm^3^ × 8 μm^3^ discretized over 50-nm steps. The simulation time resolution is set to 160 fs. The intensity distribution is calculated as the beam was focused 5 µm below the sample surface. FDTD simulations were performed with Lumerical version 2020a in the Microsoft Windows 10 operating system. The calculation was implemented on a PC equipped with Intel Core i7-11800H CPU and one NVIDIA GeForce RTX 3050Ti Laptop graphical processing unit (GPU) with a typical computing mesh accuracy of 3. As shown in Figures 4C1–C3, the dramatic distortions of the focal field distribution caused by the mismatched vertical interface can be revealed by FDTD calculations. An asymmetric double-peaked distribution close to the interface is generated due to the vertical refractive index mismatches. Traditional propagation models could not take full account of sample heterogeneity near focus, which are usually neglected in polarization-resolved SHG microscopy studies. Figure 4C4 shows the intensity line profiles along the horizontal lines in Figure 4C1 at the position of y = 250 nm. It is particularly interesting to find that there is a slight difference between the focus field distributions of left-handed and right-handed circular polarized beams.

Next, the capability of FDTD methods to model circular-polarization-excited SHG imaging in index-mismatched media in the case of a vertical interface between water and a KTP material are investigated. For the KTP material, a non-zero diagonal second-order susceptibility was considered. We speculate that index mismatch of the vertical interface results in significant profile distortions. Figure 4D1 illustrates the SHG intensity distribution when the focused beam is 2D scanned across the interface in a region of x ∈ (−3 µm, 3 µm), z ∈ (−5 µm, 3 µm). As shown in the extracted profile (Figure 4D2), for refractive index mismatch, the position of the peak or valley signals is not located at the interface, which is inconsistent with intuitive perception. The right-handed circular polarization has almost the same distribution with left-handed circular polarization. These results are important for the quantitative interpretation of SHG images of KTP growth.

## Conclusion

In summary, we have demonstrated a previously unidentified rigorous model to demonstrate circular dichroism SHG microscopy through stratified media for KTP crystal. In our proposed model, the refractive index mismatches and the imaging depth are taken into account for quantitative analysis of the CD-SHG signal. It has been demonstrated that the azimuthal angle of the peak value (SHG) or valley value (CD-SHG) is strongly related to the excitation polarization when the KTP sample is excited by different circular polarizations. Importantly, for the same excitation, the refractive index mismatches and the imaging depth also affect the azimuthal angle. Besides, the numerical framework based on FDTD can be an applicable simulation strategy to investigate CD-SHG microscopy with sample refractive index heterogeneity. It is expected that the proposed model can contribute towards new insights into nanoscale morphology of KTP and the experimental configuration optimization of CD-SHG microscopy.

## Data Availability

The raw data supporting the conclusion of this article will be made available by the authors, without undue reservation.
